# Multi-omics network model reveals key genes associated with p-coumaric acid stress response in an industrial yeast strain

**DOI:** 10.1038/s41598-022-26843-2

**Published:** 2022-12-28

**Authors:** F. E. Ciamponi, D. P. Procópio, N. F. Murad, T. T. Franco, T. O. Basso, M. M. Brandão

**Affiliations:** 1 Center for Molecular Biology and Genetic Engineering (CBMEG), State University of Campinas (Unicamp), Av. Cândido Rondon, 400, Campinas, SP 13083-875 Brazil; 2 Department of Chemical Engineering, University of São Paulo (USP), Av. Prof. Luciano Gualberto, 380, São Paulo, SP 05508-010 Brazil; 3 School of Chemical Engineering (FEQ), State University of Campinas (Unicamp), Av. Albert Einstein, 500, Campinas, SP 13083-852 Brazil

**Keywords:** Biochemical reaction networks, Gene regulatory networks, Gene expression profiling

## Abstract

The production of ethanol from lignocellulosic sources presents increasingly difficult issues for the global biofuel scenario, leading to increased production costs of current second-generation (2G) ethanol when compared to first-generation (1G) plants. Among the setbacks encountered in industrial processes, the presence of chemical inhibitors from pre-treatment processes severely hinders the potential of yeasts in producing ethanol at peak efficiency. However, some industrial yeast strains have, either naturally or artificially, higher tolerance levels to these compounds. Such is the case of *S. cerevisiae* SA-1, a Brazilian fuel ethanol industrial strain that has shown high resistance to inhibitors produced by the pre-treatment of cellulosic complexes. Our study focuses on the characterization of the transcriptomic and physiological impact of an inhibitor of this type, *p*-coumaric acid (pCA), on this strain under chemostat cultivation via RNAseq and quantitative physiological data. It was found that strain SA-1 tend to increase ethanol yield and production rate while decreasing biomass yield when exposed to pCA, in contrast to pCA-susceptible strains, which tend to decrease their ethanol yield and fermentation efficiency when exposed to this substance. This suggests increased metabolic activity linked to mitochondrial and peroxisomal processes. The transcriptomic analysis also revealed a plethora of differentially expressed genes located in co-expressed clusters that are associated with changes in biological pathways linked to biosynthetic and energetical processes. Furthermore, it was also identified 20 genes that act as interaction hubs for these clusters, while also having association with altered pathways and changes in metabolic outputs, potentially leading to the discovery of novel targets for metabolic engineering toward a more robust industrial yeast strain.

## Introduction

Expansion of the global lignocellulosic ethanol production is heading towards second-generation ethanol (2G) biorefineries. Although 2G ethanol is still more expensive than first-generation ethanol (1G), current production costs for 2G are up to 50% higher than 1G^1,2^, recent advances in biofuel technology suggest that 2G ethanol will be more cost-efficient in the long run, with some of the more optimistic scenario placing the turning point for this technology in the year 2025^1,3,4^. For commodities like ethanol, even small changes in production costs can have a big impact on the supply chain. Reducing operating costs by a few cents can result in savings of millions of dollars per year^5–7^.

During 2G ethanol production, before the biomass being placed in fermentation vats, the lignocellulosic feedstock undergoes pre-processing to unleash less complex sugars located in the cell wall to make these molecules available for the yeasts^8^. However, this process also releases several toxic compounds in the medium, and, as a result, it requires microorganisms with increasing resistance to inhibitors generated during pretreatment processes^9,10^. Therefore, understanding how these inhibitory molecules affect the fermentative performance of *Saccharomyces cerevisiae* is essential to implementing strategies to increase its robustness against adverse conditions in industrial fermentation and contributing to its implementation as a stable platform for biofuel production^11–13^.

Among these compounds, two classes of molecules—furans and organic acids—have an active physiological impact on the growth rate and overall fermentation metabolism of *S. cerevisiae*^11,13–18^. Additionally, phenolic compounds also inhibit the production of ethanol in anaerobic fermentation^13^. Although certain *S. cerevisiae* strains are resistant to these molecules, the molecular mechanism used by these yeasts to metabolize such inhibitors into less toxic compounds is complex, involving multiple regulatory processes and pathways^19^. One of the major byproducts resulting from the sugarcane bagasse pretreatment in the production of 2G bioethanol is *p*-coumaric acid (pCA)^20,21^, which was found to be in concentrations of up to 2.0 g/kg (dry weight) of bagasse after pretreatment^22,23^. This chemical usually inhibits the growth of *S. cerevisiae* and disrupts the production of ethanol^24–27^. Although some reports show that certain *S. cerevisiae* strains are capable of surviving high concentrations of pCA, or even being engineered to serve as templates for the production of this compound^28,29^, the same cannot be said for strains currently in use in the 2G bioethanol industry. These strains have a significantly different genomic makeup, in the form of nucleotide variation and structural rearrangements, caused by the intense selection process that this particular group of *S. cerevisiae* was subjected to^30–33^, and appear to be more susceptible to the inhibitory effects of these compounds^26,34,35^. Moreover, pCA is insoluble in water, but easily reacts and solubilizes in ethanol, posing a significant challenge for any type of industrial-scale fermentation process^36^.

Recently, a Brazilian industrial strain used in the bioethanol industry called SA-1 was shown to be highly resistant to several lignocellulosic inhibitors, being capable of maintaining 70% of its normal growth rate even when exposed to 7 mM of pCA, a feature that was not observed even in other industrial strains, such as the case of JAY270, a haploid derivative of PE-2 which is one of the most widespread strains currently in use in the Brazilian bioethanol industry^26,37,38^. However, the molecular characterization of specific survival mechanisms used by SA-1 to survive in such conditions has not yet been described. Considering that the response of *S. cerevisiae* gene expression to environmental conditions is a powerful tool for identifying targets associated with increased ethanol production and survivability and has been used to direct bioengineering efforts toward the desired phenotype by altering the transcriptional machinery of these organisms^39–41^. The use of data obtained for differentially expressed genes in combination with systems biology approaches also allowed researchers to identify the metabolic pathways that are affected (activated or repressed) under specific conditions^42–45^.

Our study focuses on characterizing the SA-1 strain profile when exposed to high concentrations of pCA under continuous fermentation conditions in a controlled bioreactor environment, using a combination of metabolite analysis, transcriptomics, and genomics in an integrative and systemic multi-omics analysis to elucidate the underlying mechanisms by which this particular strain is capable of thriving even when exposed to such inhibitors.

Our main objectives are not only to characterize the molecular aspects of the response but also to identify key genes associated with the response to such inhibitors, allowing a deeper understanding of these functions. By using these findings as a framework for future bioengineering efforts, be it in the form of gene models or pathways of interest, it is expected our work to provide valuable insights into the development of more robust industrial strains that are capable of increased survival rates when exposed to the adversary conditions present in industrial fermentation vats, assisting in the reduction overall production costs of 2G ethanol production and establishing this platform as a stable source for biofuel production.

## Results and discussion

### Metabolic changes in SA-1 strain upon pCA exposure

A thorough understanding of the effects of pCA on yeast metabolism is required to generate potential metabolic engineering strategies that can improve strain robustness. Since lignocellulosic hydrolysates contain a high number of phenolic compounds of which 80% represents pCA, this compost was added to the feed-medium of carbon-limited chemostats. Cultivation without inhibitors served as control. Data collected along each batch phase was linearized by applying the natural logarithm to exit of CO_2_ values as a function of time. Specific consumption rates of glucose and specific production rates of selected extracellular metabolites are shown in Table 1 and Table S1.

**Table 1:** Physiology of *S. cerevisiae* strains in glucose-limited anaerobic chemostats at a dilution rate of 0.1 h^−1^.

Conditions/parameters	Control	7 mM pCA
µ (batch phase)	0.38 ± 0.02	0.37 ± 0.03
Residual glucose (g/L)	0.69 ± 0.05	0.69 ± 0.22
*q g*lucose	− 5.80 ± 0.05	− 7.34 ± 0.50
*q C*O_2_	9.82 ± 1.01	11.04 ± 1.03
*q e*thanol	8.66 ± 0.34	13.27 ± 1.17
*q g*lycerol	1.04 ± 0.07	0.84 ± 0.24
*q l*actate	0.08 ± 0.00	0.06 ± 0.03
*q* pyruvate	0.02 ± 0.00	0.03 ± 0.02
*q a*cetate	0.00 ± 0.00	0.00 ± 0.00
X	2.64 ± 0.01	2.14 ± 0.02
Y_X/S_	0.13 ± 0.00	0.09 ± 0.00
Y_Eth/S_	0.38 ± 0.02	0.46 ± 0.01
C recovery	98.5 ± 3.3	100.7 ± 0.1

Specific rates (*q*) are given in mmol g^−1^ h^−1^, *µ* in h^−1^, yeast biomass (X) in g DW L^−1^, conversion factor of substrate into biomass (Y_X/S_) in g DW g glucose^−1^, ethanol yield (Y_*Eth*/S_) in g ethanol^−1^ g glucose^−1^, and C recovery in (%). The µ value represents the growth rate measured during the batch phase before the steady-state is achieved. Data is the average values of duplicate experiments ± deviation of the mean.

It has been reported that in aerobic cultures containing pCA, the growth rate is significantly reduced in a dose-dependent manner ⁠and inhibiting the efficient bioconversion of lignocellulose biomass by fermentative organisms^46,47^. Although the *S. cerevisiae* CEN.PK113-7D strain was shown to be capable of slow in situ catabolic conversion of 9.7 mM pCA in aerobic batch cultivations, performing a complete conversion of pCA into other phenolic compounds over a period of 72 h^46,47^. Our results indicate that under anaerobic conditions, the same catabolic effect does not occur with the pCA concentrations remaining relatively stable across steady-state measurements. This highlights the importance of analyzing industrial *S. cerevisiae* strains under anaerobic chemostat conditions, especially considering that mitochondrial respiration has been shown to be of significant importance for controlling yeast growth rate processes and resistance to phenolic compounds^48–50^.

**Figure 1:**
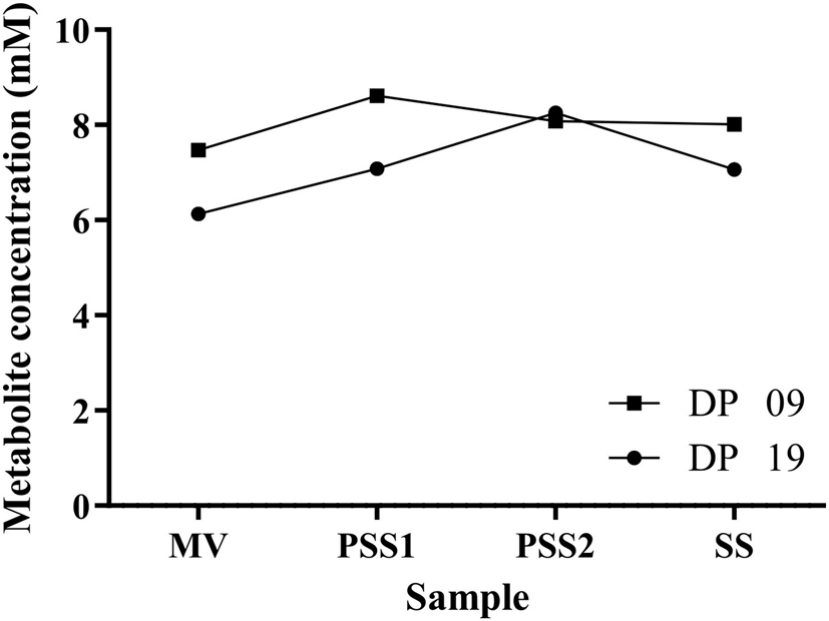
pCA concentration during anaerobic glucose-limited chemostat cultivations of *S. cerevisiae* SA-1 strain. *MV* medium vessel, *PSS1* first pre-steady-state, *PSS2* second pre-steady state; SS, steady-state. DP09 and DP19 code for the duplicate runs. The pre-steady-state samples (PSS1 and PSS2) represent samplings at 24 and 48 h after starting the feeding, respectively.

Exploring the differences in the physiological parameters resulting from the addition of the phenolic compound, multiple alterations were observed. Some specific consumption and production rates increased, such as for glucose (26%), CO_2_ (12%), and ethanol (53%). On the other hand, decrease in biomass yield (22%), and the glycerol production rate (19%) was observed (Table 1). In anaerobic glucose-limited chemostat cultures of the *S. cerevisiae* strains, carbon is mainly diverted to ethanol and CO_2_, and minor amounts of glycerol, lactic and acetic acids, with a concomitant formation of yeast biomass. The ethanol yield of SA-1 in the control condition was 21% lower than in the presence of pCA. Moreover, under anaerobic glucose-limited chemostat cultivations pCA is not metabolized by SA-1 (Fig. 1).

Overall, physiological data collected from the anaerobic chemostat cultures showed that pCA exposure may increase ethanol production in SA-1 strain (log2(foldChange) = 0.58; t-test p-value < 0.05). These changes are accompanied by an increased glucose uptake rate (log2(fC) = 0.33, pval = 0.10), which might suggest alterations in the metabolic state. In addition, these changes were followed by a decrease in the overall dry-weight cell biomass (log2(fC) =  − 0.27, pval < 0.05) and biomass yield (log2(fC) = − 0.25, pval = 0.10). This is different from previous studies that showed that *S. cerevisiae* Ethanol Red^®^, when exposed to pCA during aerobic batch cultivations, increases biomass yield and decreases ethanol yield in comparison to control conditions (without pCA)^47^⁠. Strains resistant to lignocellulosic inhibitors have lower growth rates when exposed to such compounds^27^⁠; however, SA-1 strain showed no signs of slower growth during the batch phase, with both control and treated samples showing an average growth rate around 0.37 h^-1^.

### Differential gene expression between treated and control samples

Gene expression analysis based on RNASeq data revealed that both conditions (treated and control) have a high correlation between biological duplicates (Pearson’s R^2^ > 0.99, Fig. S1). These values are within the established parameters for chemostat cultures^51^⁠, which generate replicates with low biological variability. Additionally, the principal component analysis showed that 79.57% of the explained variance observed in the samples can be associated with the axis that represents separation based on experimental conditions, with only 10.28% of explained variance being associated with alterations from samples under the same conditions (Fig. S1B). These analyses indicate significant changes in the transcriptomic landscape of SA-1 strain when exposed to pCA.

In order to assess the quality of our differential gene expression data, we measured the observed relationships between average gene expression ratios and their deviation from the mean (Fig. S1C), the observed p-values and variation and expression quantiles (Fig. S1D) as well as the correlation between observed gene expression standard deviation and median expression value (Fig. S1E). Not only does our quality control data show that there is little-to-no bias in our dataset, it has a similar behavior to what is expected from a well controlled differential gene expression experiment when using RNA samples collected in the second steady-state of chemostat cultures^52^.

**Figure 2:**
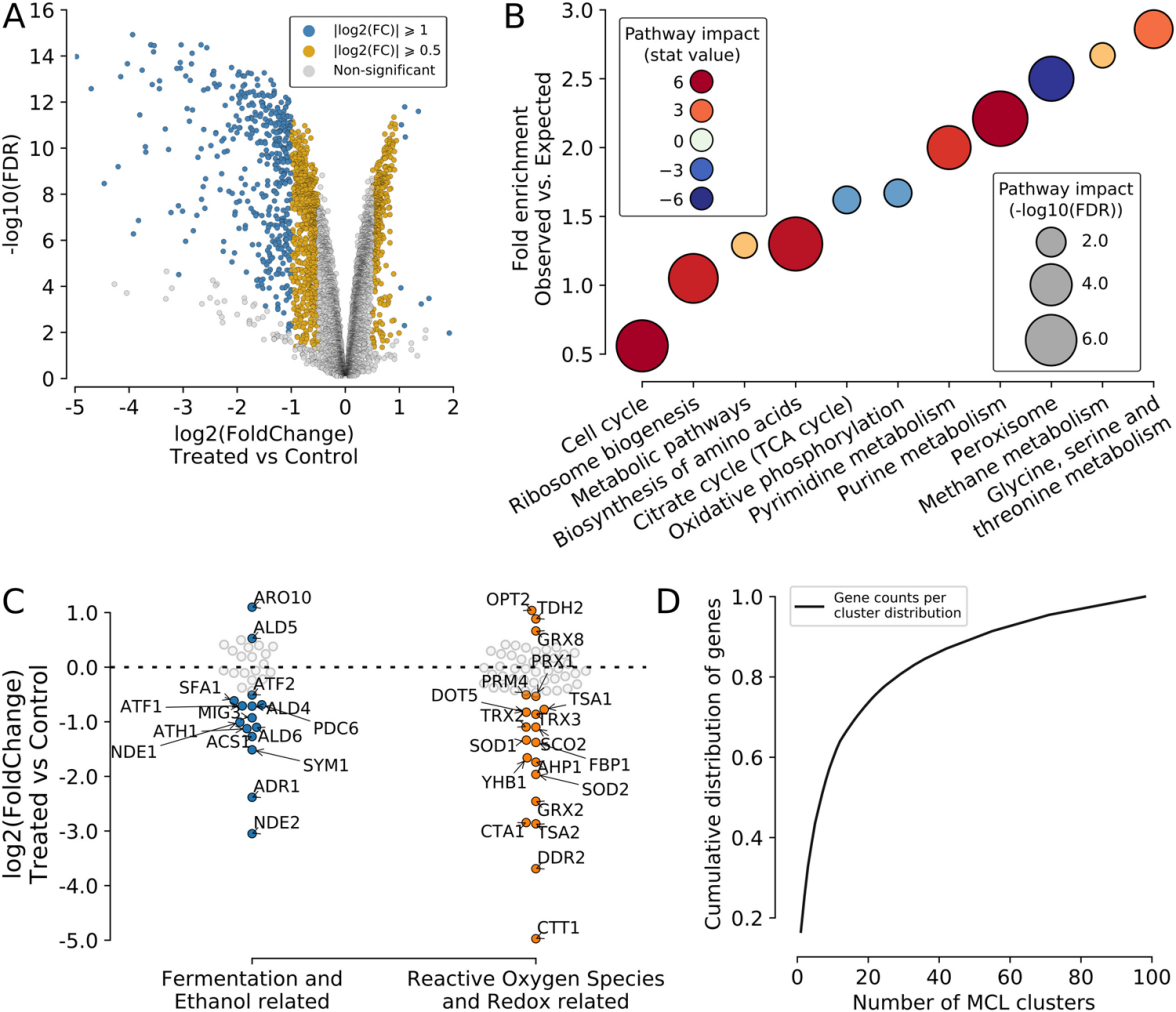
Differentially expressed genes under *p*-coumaric stress are skewed towards downregulation and can be grouped into functional clusters. (**A**) Volcano plot showing the relation between log2(Fold Change) (X-axis) and − log10(FDR) (Y-axis) for differentially expressed genes (DEGs in blue and gold) after pCA treatment. (**B**) Point plot showing the predicted perturbations in KEGG pathways (X-axis) based on RNASeq data. The Y-axis shows the fold-enrichment score identified for each pathway of DEGs; the size of the point shows the significant value of the prediction (in − log10(FDR) scale), and the color represents the direction of perturbation: downregulated (brown) or upregulated (blue). (**C**) Swarmplot showing the log2(FoldChange) profile (Y-axis) of the 13 DEGs associated with fermentation and/or ethanol (blue) and the 17 DEGs associated with reactive oxygen species response and redox processes (orange). (**D**) Cumulative distribution function (CDF) plot showing the percentage of DEGs (Y-axis) that are in clusters (X-axis), starting from the biggest clusters (in the number of genes).

It was identified a total of 1472 differentially expressed genes between conditions (404 up- and 1068 down-regulated); however, only 448 (10 up- and 438 down-regulated) of the genes was statistically significant, with p-value < 0.05, FDR ≤ 0.01, and had |log2(FoldChange)| ≥ 1 (Fig. 2A, Table S2). It is known that the presence of insoluble lignocellulosic inhibitors in the medium can promote generalized downregulation of gene expression in another industrial *S. cerevisiae* strain^34^. With that in mind, the possible transcriptomic alterations induced by pCA in our chemostat experiments were explored. By using gene expression data obtained from our RNA-seq data, we observed a tendency towards global downregulation of the transcriptional machinery. This same expression pattern was already observed by Moreno et al.^34^ as a response to the presence of insoluble inhibitory compounds in the fermentation medium. Our findings not only reflect the same pattern reported, but highlights the importance of understanding the biological impacts of different types of inhibitory conditions on fermentative microorganisms for directing bioengineering efforts towards more robust strains for the ethanol industry.

The DEGs identified in our study were mapped in the KEGG database^53^⁠ to identify perturbations in pathways containing SA-1 differentially expressed gene sets (either up- or down-regulated), which affect the overall pathway activity (Fig. 2B, Table S3). Using this approach, a total of 11 pathways that had statistically significant gene set alterations by pCA stress were identified, and three were negatively altered (repressed): oxidative phosphorylation, citrate cycle, and peroxisome. The other eight had perturbations with positive effects on the pathway (activated): cell cycle, ribosome biogenesis, metabolic pathways, biosynthesis of amino acids, pyrimidine metabolism, purine metabolism, methane metabolism and glycine, serine, and threonine metabolism. Additionally, a total of 20 differentially expressed genes (17 downregulated and 3 upregulated) were identified, associated with redox regulation and/or response to reactive oxygen species and another set of 15 DEGs (13 downregulated and 2 upregulated) that can be linked to ethanol metabolism and/or fermentation (Fig. 2C). No overlapping genes between these two sets (ROS/Redox and Ethanol/Fermentation) were observed.

Amongst the impacted pathways, purine metabolism was one of the most prominent, this pathway is involved in the formation of adenine and guanine. The former is an essential part of the overall cell metabolism, in the form of ATP/ADP/AMP, by providing energy for cellular processes, and the latter plays a distinct role in cell response to stress conditions in the form of GTP/GDP, a molecule often used in signaling processes during stress response for transcriptional regulation^54^ and glucose signaling via GPCR^55^. The increase in the metabolism of these compounds could also be related to an increased rate of metabolic processes during the stress response. Furthermore, several differentially expressed genes that have known associations, via Gene Ontology^56,57^, to reactive oxygen species and ethanol fermentative processes are identified. Both of these processes are known to be intricately related to the mitochondria and peroxisome organelles^48,58,59^, and are known hallmarks of yeast response to stress induced by lignocellulosic inhibitors^59–62^. In addition to the aforementioned processes, it was also identified several other upregulated pathways that are linked to mitochondrial activity, such as biosynthesis and metabolism of amino acids^63^. These processes are intrinsically associated with the TCA cycle, which is downregulated in our dataset, and of paramount importance in maintaining amino-acid homeostasis, which, in turn, is vital for promoting long-term viability in yeasts^64^.

In addition to several of our results suggesting alterations surrounding pathways related to the mitochondria in response to pCA stress, multiple studies already pointed out the importance of this organelle towards the resistance to this particular compound^65–67^. However, the exact mechanisms by which the pCA affects this *S. cerevisiae* organelle under anaerobic conditions are still largely unexplored. When exploring the literature available for other organisms, some studies conducted in rat liver and human cells showed that pCA may cause damage to the mitochondria: by inhibiting the pyruvate transport mechanism^68,69^, inducing reactive oxygen species (ROS) damage^70^⁠ and mitochondrial membrane depolarization^71^⁠. Another study demonstrated that PAD1, a mitochondrial protein that is downregulated (log2(FC) =  − 0.5) in SA-1, is essential for the decarboxylation of phenylacrylic acids^65^. In all the three cases described in the literature, mitochondrial damage ultimately leads to a signaling cascade that starts cell autophagy.

Graph network clustering using known protein–protein interaction data associated with fold changes derived from RNASeq was applied to identify co-expressed gene clusters. This approach is advantageous for inferring networks from data with a low number of samples, as is the case of most experiments in biology. This is because it brings precision from a prior knowledge of a manually curated database (STRINGdb) combined with fold change values that include the particularity of the experiment. A total of 98 clusters were found within the differentially expressed genes, with 50% of DEGs being located in the 7 biggest clusters (Fig. 2D, Table S4). These clusters were then characterized according to their expression profile (Table S5), based on the distribution of log2(fold change) for genes within each cluster, enrichment of significant Gene Ontology classes and KEGG pathways (Table S5). Additionally, no correlation (R^2^ = 0.033) was found between the number of genes and the overall standard deviation of fold changes observed within each cluster.

### Functional characterization of co-expressed gene clusters

**Figure 3:**
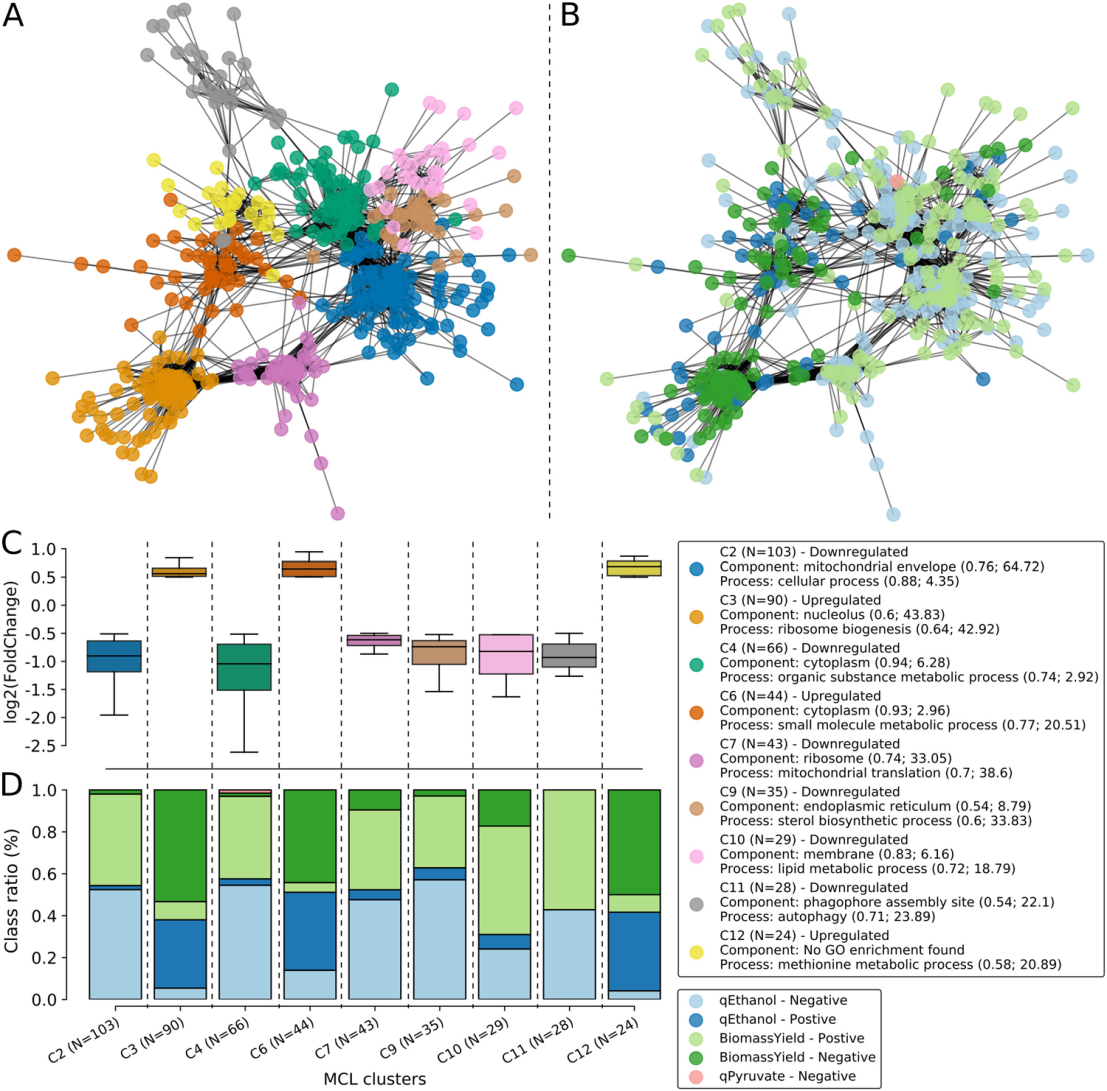
Co-expressed gene clusters can be classified according to functional enrichment and association with phenotype alterations. (**A**, **B**) Network representation of the 9 co-expressed gene clusters selected for further exploration. On the right (**A**), the clusters were classified according to their gene ontology functional enrichment; on the left (**B**) each gene in the network was classified according to their probability of having a positive or negative association with either qEthanol or biomass yield metabolic phenotypes. (**C**) Boxplot showing the expression profile (Y-axis) of each of the selected clusters (X-axis). The color of each cluster matches those of the network. (**D**) Stacked barplot showing the ratio of genes (Y-axis) associated with the metabolic classes (positive/negative relation to biomass yield/qEthanol) for each of the selected clusters.

To further explore the clustered gene sets, the clusters were filtered bythe fold change values, located in a distance of 1.5 × IQR (interquartile range), were in the same quadrant, either above or below zero, and had more than 20 genes. A total of 9 clusters (C2, C3, C4, C6, C7, C9, C10, C11, and C12), with a total of 462 DEGs, were selected and characterized according to biological functional enrichment and association (Fig. 3A, Table S6) with phenotypic alterations (Fig. 3B, Table S7). Six of these clusters (C2, C4, C7, C9, C10, and C11) comprised downregulated genes, while 3 (C3, C6, and C12) were mostly from upregulated genes (Fig. 3C). However, each cluster had distinct associations with biomass yield and ethanol production (Fig. 3D). Downregulated clusters tended to share positive associations with biomass and negative relations with ethanol production. Upregulated clusters, on the other hand, showed an inverse pattern, with negative regulation of biomass and positive association with ethanol.

To delve deeper into the transcriptomic landscape alterations induced by pCA stress on SA-1 strain, we shifted our focus to the co-expressed gene clusters extracted from our network in order to characterize which functional groups of genes were being up/down-regulated and if those clusters could be related to the pathways changes observed previously. Amongst the groups of genes that stood out from the background, it was noticed that one of the co-expressed clusters associated with autophagy (C11) was actively repressed in SA-1 under p-coumaric stress. Our data also suggests a potential disruption of the mitochondria, with 70% of the genes located in two downregulated clusters being directly associated with mitochondrial cellular processes (C2) and translation (C7). This correlates with the negative impact observed for KEGG pathways associated with the citrate cycle and oxidative phosphorylation. Furthermore, 20 of the 27 differentially expressed genes associated with the peroxisomal pathway (AGX1, CAT2, CTA1, DCI1, ECI1, FAA2, IDP2, IDP3, PEX1, PEX11, PEX14, PEX2, PEX5, POT1, POX1, PXA1, PXA2, SPS19, YAT1, and YAT2) are also located in a downregulated cluster associated with the cytoplasmic organic substance metabolic process (C4). Peroxisomes are involved with long fatty acid degradation and biosynthesis in yeasts^72,73^, with lipid metabolism/biogenesis being one of the downregulated gene clusters identified (C10). In contrast, increased expression was detected in genes linked with nuclear ribogenesis (C3) and metabolic activities (C6 and C12). When compared with the predicted pathway impact, C3 has 19 of the 23 DEGs associated with the ribosome biogenesis pathway (AFG2, DIP2, EMG1, KRE33, POP1, POP7, PWP2, RIO2, RIX7, RNT1, SDO1, UTP13, UTP18, UTP22, UTP4, UTP5, UTP6, UTP8, UTP9), C6 contains 14 of the 31 DEGs associated with purine metabolism (ADE1, ADE12, ADE13, ADE17, ADE2, ADE4, ADE5,7, ADE6, ADE8, ADK1, GUD1, HPT1, IMD2, and IMD4) and C12 contains 13 of the 31 DEGs associated with the biosynthesis of amino acids (ARG1, ASN1, GLY1, HIS1, HIS4, HIS6, HIS7, HOM3, LYS2, LYS4, SER1, SER2, and SHM2). When coupled with the alterations observed in gene sets associated with the mitochondria, our data imply that multiple biological pathways are being regulated to compensate for the stress induced by pCA exposure. Such regulation might be associated with processes used by SA-1 to survive under adverse conditions.

**Figure 4:**
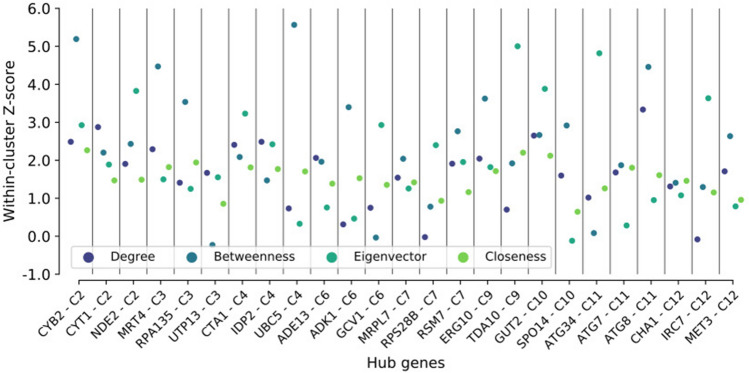
Hub genes are located in co-expressed gene clusters. Swarmplot showing the centrality scores (Y-axis) measured for the hub-genes selected from each cluster (X-axis). Each metric used is shown in a different color.

To further explore the characterized clusters, we also evaluated each of the genes found within the clusters to establish which of them acted as “network hubs” in their respective clusters, based on their values for eigenvector centrality, betweenness, degree, and closeness. In total, 25 genes (Fig. 4, Table S8) that act as the main points of interaction for the genes in the network were identified.

### Prediction of genomic short-variants based on RNA-seq

**Figure 5:**
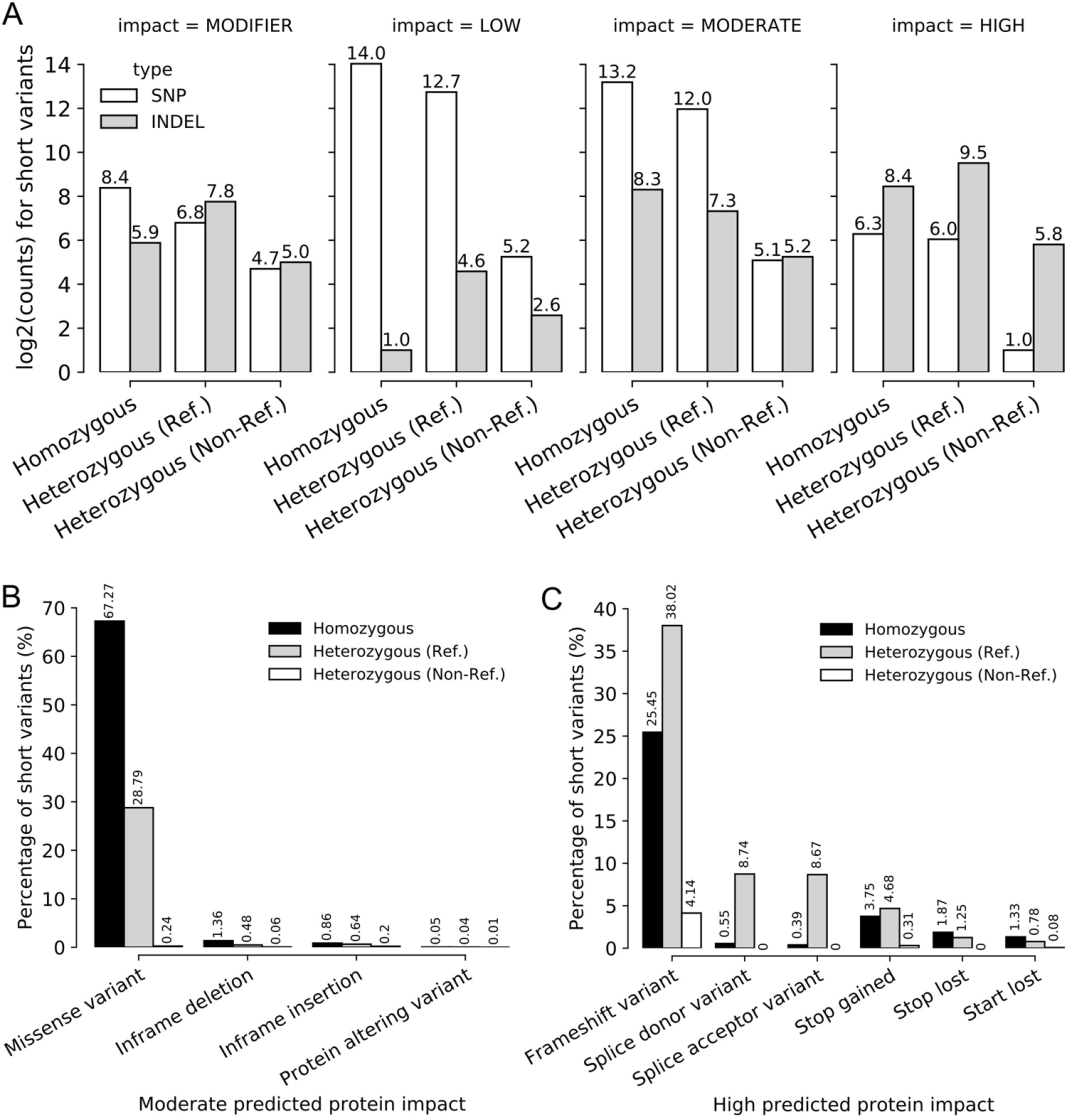
Homozygous missense and heterozygous frameshift variants are the major classes of short-variants with predicted moderate-to-high protein impact. (**A**) Barplots showing the overall number of variants (Y-axis) identified from RNASeq data for the SA-1 strain when compared to the R64-1-1 reference annotation for *S. cerevisiae*. The X-axis shows the ploidy identified for each variant: homozygous, heterozygous with reference, or heterozygous without reference. The columns are separated according to the overall level of predicted protein impact, from lowest to highest. (**B**, **C**) Barplot showing the breakdown of the predicted protein impacts (X-axis) and their associated proportions (Y-axis) for variants with moderate (**B**) and high (**C**) predicted protein impact.

Using data collected from high-throughput RNA sequencing, we reconstructed short variants (SNPs and INDELs) that occur within the transcripts⁠ and predicted the impact that they might have on their associated coding sequence^74^. A total of 38,420 short variations were identified (compared to the R64-1-1 reference annotation) that can be subdivided into three major categories: homozygous (both alleles carry the variant), heterozygous with reference (one allele carries the variant, while the other is equal to the reference) and heterozygous without reference (both alleles carry different variants, and neither is equal to the reference). These short variants were then classified according to their predicted impact on their associated coding sequence (Fig. 5A): 683 modifier variants (non-coding, e.g., intronic or UTR variants); 23,388 low impact variants (e.g., synonymous mutations); 13,655 moderate impact variants (e.g., missense mutations that preserve overall protein length/structure) and 1093 high-impact variants (e.g., frameshift INDELs or stop-gaining SNPs). However, the sum of the subclasses exceeds the total of variants (Table S9). This occurs because a variant can impact multiple genes, and current limitations make it difficult to solve such conflicts using HTS data alone^74,75^.

The major classes of high-impact variants involved frameshift variants, and most of them (~ 38%) were heterozygous (concerning the reference) in nature. Homozygous frameshift variants also comprised the second biggest class, with approximately 25% of variants falling in that class, and non-reference heterozygous frameshifts comprising ~ 4% of high-impact variants. Heterozygotic variants located in splicing sites were also a major class of high-impact predictions, with a combined total of ~ 17%. Lastly, stop-gaining mutations represented ~ 8% of all high-impact variants, with approximately half (3.75%) being homozygotic in nature and the remaining (4.68%) being heterozygous.

Then only the variants that had predicted moderate and high protein impacts were filtered for further exploration. Our analysis showed that the major class of moderate impact alterations was the class of missense variants, with ~ 67% of them being homozygous in nature and ~ 29% being heterozygous with the reference (Fig. 5B). However, when comparing the same results for high-impact variants, a much broader distribution of CDS consequences (χ^2^ (3, N = 15,166) = 7096.5, p < 0.001, Fig. 5C) was observed.

In addition to gene expression changes, mutations are also a major player in the process of generating resistant strains for biofuel production, be them artificially generated or naturally selected^76–78^. These structural alterations can promote changes in the function of genes and proteins in multiple ways^79^, with even small mutations possibly having far-reaching effects^80,81^. By characterizing the profile of single nucleotide variants when compared to the S288C reference strain (which has extensive functional gene annotation data), it was possible to predict the impacts of mutations found in SA-1’s genes^82^. Although we recognize that a comprehensive analysis based on comparative genomics of industrial yeasts, which is outside the scope of the present article, would be ideal to characterize the genomic complexity of the SA-1 industrial strain, it's expect that, the extraction of the information on the genetic diversity encountered in SA-1 will provide an important layer of information on the mechanisms associated to pCA response on this strain.

### Assembly of a multi-omics network model for pCA response

To generate a single model that represents the association between differentially expressed genes located in perturbed pathways, the association with fermentation/ethanol and ROS/Redox, the presence of high-impact short variants, and phenotype impact prediction, all the information described in the previous sections was converted into a multi-omics graph-based network model. Integrative approaches, such as this, are especially relevant to “Big Data” datasets, such as ours, to extract comprehensive models that capture subtleties involved in biological regulation that otherwise would be lost if each “omic” was only analyzed independently^83^. This type of analysis has already been successfully used in multiple fields, from biomedical research^84,85^ to biotechnology^86,87^. Furthermore, it has been demonstrated that in-silico modeling of complex biological networks can be a powerful tool for driving the improvement of commercially relevant organisms, such as the development of an improved model of Aspergillus nidulans metabolic network models, which is important for the construction and optimization of glucoamylase-producing strains^88–90^.

**Figure 6:**
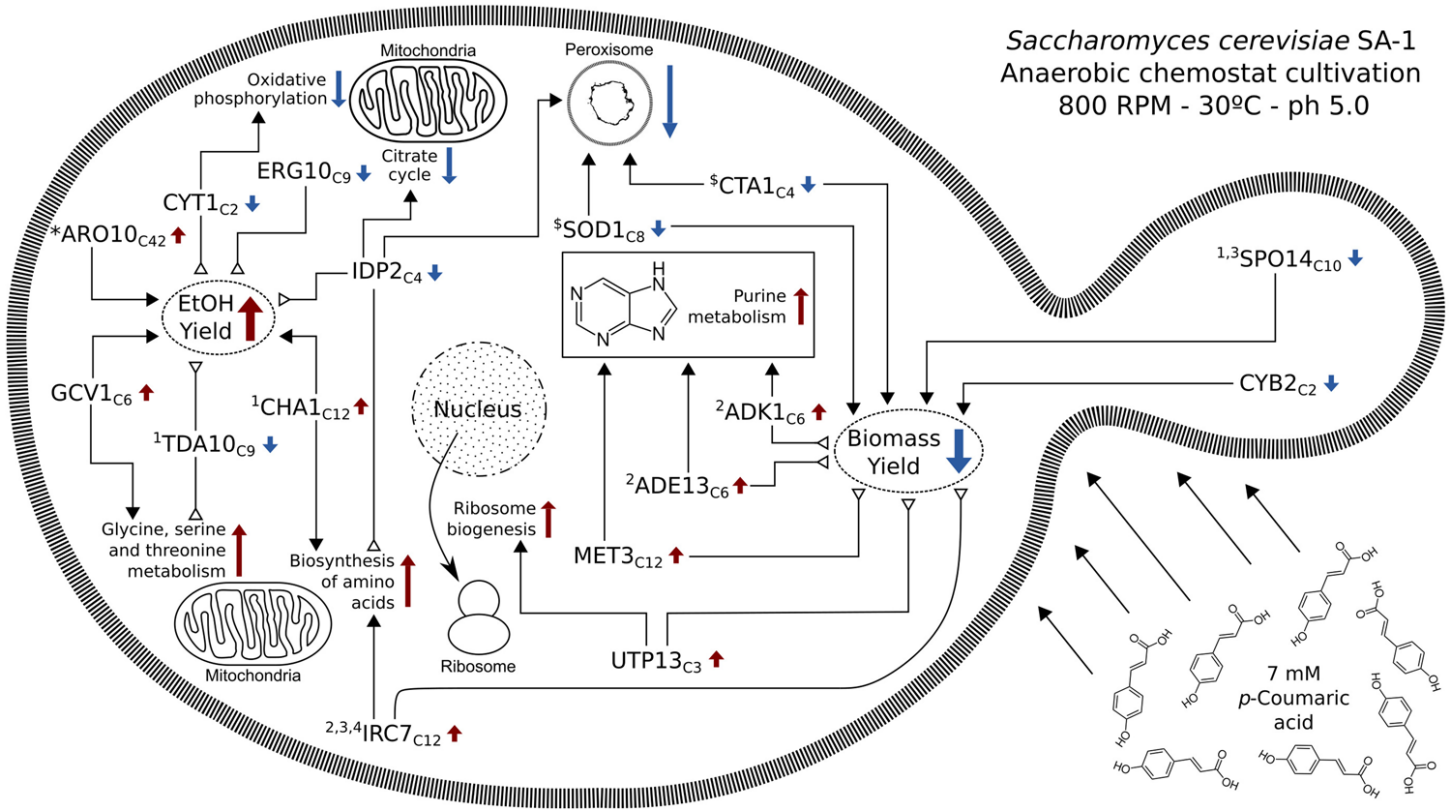
Multi-omics integrated model for alterations induced by pCA stress in the *S. cerevisiae* SA-1 strain. Network representation of the relation between hub genes, short variants (SVs), perturbed pathways, and phenotypic alterations. Red arrows indicate positive regulation (upregulated genes and pathways), blue arrows show negative relations (downregulated genes and pathways) and black arrows represent additional information sources (presence of SVs or gene ontology categories), with their connector showing the relationship as direct (solid arrowhead, both elements moving in the same direction) or inverted (hollow triangle, elements moving in opposite directions). Genes labeled with numbers indicate the presence of a short variant (1: missense homozygous mutation; 2: missense heterozygous; 3: frameshift heterozygous and 4: stop-gained heterozygous). Genes labeled with symbols represent known associations to either fermentation processes (*) or response to reactive oxygen species ($).

When applied specifically to *S. cerevisiae*, this strategy has also been proven to be crucial in unraveling novel molecular mechanisms associated with gene regulation^91^, stress tolerance^92^ and selection of targets for bioengineering^93^. From the complete network, all the edges in which at least one vertex was either a hub gene (as shown in Fig. 4) or a gene associated with fermentation/ethanol or ROS/Redox (as shown in Fig. 2C) were extracted. A total of 16 genes (Fig. 6, Table 2) were selected based on the aforementioned criteria for constructing the model, while these targets were clustered into two major groups: those associated with ethanol production (IDP2, ERG10, CYT1, ARO10, GCV1, TDA10, and CHA1) and those related to biomass yield (SOD1, CTA1, IRC7, SPO14, UTP13, CYB2, MET3, ADK1, and ADE13). In order to facilitate the discussion, we will be focusing on the hub genes mentioned previously, since they are the most representative targets for their respective co-expressed clusters. However, we strongly encourage the exploration of our entire network model made available in the supplementary files (Table S10) by the readers.

**Table 2:** List of genes that were used as nodes in the multi-omics model.

Gene	Cluster	Phenotype	Pathway	SNV	Description (UniProt keyword)
UTP13	C3	Biomass (−)	Ribosome biogenesis (+)	No	Nucleus, repeat, ribonucleoprotein, ribosome biogenesis, WD repeat, rRNA processing
ADE13	C6	Biomass (−)	Purine metabolism (+)	Yes	Isopeptide bond, lyase, purine biosynthesis, Ubl conjugation
ADK1	C6	Biomass (−)	Purine metabolism (+)	Yes	ATP-binding, acetylation, cytoplasm, kinase, mitochondrion, nucleotide-binding, transferase
IRC7	C12	Biomass (−)	Biosynthesis of amino acids (+)	Yes	Amino-acid biosynthesis, lyase, methionine biosynthesis, pyridoxal phosphate
MET3	C12	Biomass (−)	Purine metabolism (+)	No	ATP-binding, amino-acid biosynthesis, cysteine biosynthesis, cytoplasm, methionine biosynthesis, nucleotide-binding, nucleotidyltransferase, transferase
CYB2	C2	Biomass (+)	None	No	Electron transport, FMN, flavoprotein, heme, iron, metal-binding, mitochondrion, oxidoreductase, respiratory chain, transit peptide, transport
CTA1	C4	Biomass (+)	Peroxisome (+)	No	Acetylation, heme, hydrogen peroxide, iron, metal-binding, oxidoreductase, peroxidase, peroxisome
SOD1	C8	Biomass (+)	Peroxisome (+)	No	Antioxidant, copper, cytoplasm, disulfide bond, isopeptide bond, metal-binding, mitochondrion, oxidoreductase, phosphoprotein, ubl conjugation, zinc
SPO14	C10	Biomass (+)	None	Yes	Acetylation, hydrolase, lipid degradation, lipid metabolism, meiosis, phosphoprotein, repeat, sporulation
CYT1	C2	Ethanol (−)	Oxidative phosphorylation (+)	No	Electron transport, heme, iron, membrane, metal-binding, mitochondrion, mitochondrion inner membrane, respiratory chain, transit peptide, translocase, transmembrane, transmembrane helix, transport
IDP2	C4	Ethanol (−)	Peroxisome (+), Citrate cycle (+), Biosynthesis of amino acids (−)	No	Cytoplasm, glyoxylate bypass, magnesium, manganese, metal-binding, NADP, oxidoreductase, tricarboxylic acid cycle
ERG10	C9	Ethanol (−)	None	No	Acetylation, acyltransferase, cytoplasm, metal-binding, potassium, transferase
TDA10	C9	Ethanol (−)	Glycine, serine and threonine metabolism (−)	Yes	ATP-binding, cytoplasm, kinase, nucleotide-binding, nucleus, transferase
GCV1	C6	Ethanol (+)	Glycine, serine and threonine metabolism (+)	No	Aminotransferase, mitochondrion, transferase, transit peptide
CHA1	C12	Ethanol (+)	Biosynthesis of amino acids (+)	yes	Acetylation, lyase, mitochondrion, pyridoxal phosphate
ARO10	C42	Ethanol (+)	None	No	Branched-chain amino acid catabolism, cytoplasm, decarboxylase, isopeptide bond, lyase, magnesium, metal-binding, phenylalanine catabolism, thiamine pyrophosphate, tryptophan catabolism, tyrosine catabolism, ubl conjugation

This multi-level network also showed the type of interaction between genes and their targeted phenotype and associated pathway, which can be either a direct relationship between the changes in gene expression and the pathway/phenotype alteration (e.g. both upregulated) or an inverse relationship (where one is upregulated and the other is downregulated). In total, 19 positive interactions (where the gene foldChange occurs in the same direction as the change in pathway activity or metabolite measurement) and 11 negative interactions (where the gene foldChange occurs in the opposite direction as the change in pathway activity or metabolite measurement) were found. The proposed network showed 6 genes with predicted mutations (TDA10, CHA1, SPO14, IRC7, ADK1 and ADE13), 2 genes associated with ROS/Redox processes (CTA1 and SOD1) and 1 gene directly related to fermentation/ethanol (ARO10).

Genes identified as anchor nodes in the multi-omics model were annotated using keywords assigned by the UniProt database that reflect their functional and structural characteristics. The additional columns also show their predicted phenotypic association (Phenotype), any associated pathways (Pathway) and if the gene contains an SNV site (SNV).

By anchoring our network on genes and using their associations (positive, negative or neutral) to pathways, phenotypes and genomic variants, it was possible to identify two main groups of targets: those associated with ethanol production and those associated with biomass yield. Each of these groups has unique features, particularly in terms of their target pathways, which will be explored separately.

When analyzing the first group of genes (ethanol related) of the integrated response model, the TDA10 gene, an ATP-binding protein with unknown function that resembles E. coli kinases^94,95^, was downregulated (log2FoldChange − 0.72) and had an inverse relation with glycine, serine, and threonine metabolism and ethanol production. In addition, the TDA10 gene was also the target of a homozygous missense variant in position 343 of the CDS, which changes the corresponding amino acid from phenylalanine to leucine, causing structural changes to the overall protein. A negative correlation with ethanol production for the ERG10 and IDP2 genes was observed. The former (ERG10) may act in the oxidative stress response^95^ and its deletion was associated with slower doubling times and susceptibility to high NaCl concentrations^96^, being a major target for genetic engineering approaches^29,97,98^. The latter (IDP2) is an isocitrate dehydrogenase that was downregulated in our dataset (log2FC − 3.57) and has been linked to small reductions in yeast lifespan^99^—this gene was also downregulated in mutants susceptible to thermosensitive autolysis and associated with mitochondrial dysfunction^100^.

It was also identified 3 genes that had a positive correlation with ethanol production: ARO10, GCV1, and CHA1 (Fig. 2C). Besides its regulatory role in fermentation^101^, ARO10 acts in the detoxification of damaged amino acids and resistance to lignocellulosic compounds, such as HMF and furfural^102^. This gene was upregulated (log2FoldChange 1.09) upon exposure to 7 mM of pCA, with a positive correlation to ethanol production. The GCV1 gene, upregulated in our dataset (log2FC 0.82), encodes the T subunit of the mitochondrial glycine decarboxylase system and increases in expression under multiple types of stress responses in *S. cerevisiae*^103–106^; however, the exact role of GCV1 in these scenarios is still not fully understood. Lastly, the CHA1 was slightly upregulated in our dataset (log2FC 0.51) and had a positive correlation with the biosynthesis of amino acids and metabolism of glycine, serine, and threonine. This gene catalyzes the degradation of L-serine and L-threonine to use them as nitrogen sources and is upregulated in the response to ethanol stress^107^ and congo red^108^.

Both CYB2 and CYT1 are mitochondrial genes that are regulated during changes in the anaerobic metabolic processes and fermentation of glucose^109–111^. In our dataset, both genes showed a negative correlation with metabolic pathways (that is, they were downregulated while the pathway was activated). Moreover, they appear to have inverse relations with phenotypic changes: CYB2 has a positive (direct) relation with biomass yield, and CYT1 has a negative (inverse) relation with the ethanol output.

In the second group of genes (biomass related) a total of four genes (SOD1, CTA1, CYB2, and SPO14) with a positive influence on the biomass yield and five with a negative correlation (IRC7, UTP13, MET3, ADK1 and ADE13) were identified. One of the most interesting targets in this group is SOD1, a downregulated gene (log2FC − 1.34): it encodes a Cu–Zn superoxide dismutase that has the main role of catalyzing the breakdown of toxic superoxides in the cell^112^ and is also involved in signaling processes involving oxygen and glucose stimuli^113^. However, recent studies suggested that the main biological role of these proteins in yeasts is the peroxide signaling and activation of peroxisomes and multiple cell homeostasis pathways^114^. This is in accordance with our findings for both gene expression, pathway impact and co-expressed gene clusters enrichment. The other gene associated with response to reactive oxygen species was CTA1, a downregulated gene (log2FC − 2.84) in our dataset. This gene encodes a catalase associated with ROS detoxification in peroxisome and in the mitochondria^115^, and its activity is relevant in oxidative^116^, acetic acid^117^ and heat^118^ stress responses. These genes showed positive associations with the activity of the peroxisomal pathway, which is one of the most affected by the stress induced by pCA in SA-1 strain. While SOD1 did not appear as a hub in the selected gene clusters, it did act as a major interactor for cluster 8, which is associated with regulatory and cell homeostasis pathways (Table S5). However, CTA1 is a major interaction hub for C4, being enriched for genes related to the metabolism of organic substances in the cytoplasm. Additionally, the SPO14 (log2FC − 0.82) gene was associated with changes in the cell cycle regulation (Honigberg et al. 1992) and regulation of lipid metabolism^119,120^ in *S. cerevisiae*. In addition to its role in the cell cycle, SPO14 was also a hub gene for cluster 10, which is enriched in genes for the lipid metabolic process.

As for the genes that had negative correlations with the biomass yield, the IRC7 gene seems to be a target with multiple associated conditions. Besides its transcriptional behavior (log2FC 0.60) in SA-1 strain, when exposed to pCA, this gene is involved in the production of thiol compounds^121^ and yeast survivability using cysteine as nitrogen source^122^. This gene was the most affected by our analysis of variants, accumulating a large amount of moderate-to-high-impact impact variants in heterozygosity, but none in homozygosity, suggesting that one of the alleles might be severely impaired. This corroborates an analysis of wine fermenting yeasts, which showed that several *S. cerevisiae* strains carried inactivating mutations for one or both alleles of IRC7^123^, reducing the overall enzymatic activity of this protein. Another study showed that the over-expression of IRC7 also resulted in the increased production of hydrogen sulfide^122^, a volatile sulfur compound that has been linked to increased longevity in *S. cerevisiae*^124^⁠. Lastly, 3 genes that showed a positive correlation with purine metabolism: MET3 (log2FC 0.73), ADK1 (log2FC 0.50) and ADE13 (log2FC 0.82) were also identified. Upregulated in our dataset (log2FoldChange 0.73), MET3 is an ATP sulfurylase involved in sulfate and methionine metabolism^125^, which was upregulated during hypoxia^48^. Moreover, the over-expression of ADE13 may increase fermentation efficiency under acetic acid stress^126^, while ADK1 appears to be activated in response to sulphuric acid^127^ and to heat stress^128^. These three genes were also associated with clusters enriched in genes linked to the regulation of metabolic processes.

## Conclusion

Our results suggest that *p*-coumaric acid (pCA) stress may induce higher cellular activity in SA-1 strain under anaerobic conditions, with increased glucose uptake rate, and CO_2_ and ethanol production rates, being the major indicators obtained from quantitative physiological data. In accordance, it was also observed a decrease in biomass yield and overall dry-weight cell biomass, which implicates the existence of some type of disturbance in the cell homeostasis. It was demonstrated that pCA stress can cause an overall activation of metabolic and biosynthesis pathways, which are also followed by increased rRNA biogenesis. Downregulation of several mitochondrial and peroxisomal-associated pathways may also be an indicator of cellular damage caused by the exposure to pCA; our data suggest that SA-1 strain has yet-to-be-explored molecular mechanisms that allow them to circumvent triggers that lead to programmed cell death. At the gene level, multiple genes that could be novel and/or interesting targets for bioengineering were identified. Our results highlight the importance of an integrated approach for target identification and association with phenotypes of interest for industrial applications. By using network-enhanced gene cluster detection, genes that could be the most influential in their biological vicinity were identified. These “hub genes'' are prime targets for genetic engineering approaches, as they are the ones with the highest impact on their sphere of influence and are most-likely to produce deep alterations in the associated biological process within the gene community. Although exploratory in nature, the data presented in this study contributes to understanding the characteristics of pCA-induced stress in *S. cerevisiae* and deepening the knowledge of mechanisms used by industrial yeast strains that can thrive under high-stress conditions, such as exposure to lignocellulosic inhibitors.

Taken together, our results show that the biological mechanisms used by *S. cerevisiae* SA-1 to survive under the influence of lignocellulosic inhibitors are much more intricate than previously understood. Multiple biological pathways, which sometimes have opposite effects when analyzed individually, are intertwined in a complex balance that allows these yeasts to thrive even when exposed to high levels of stress. Systemic analysis is essential to understand the nuances involved in such interactions, with several information sources and analyses being integrated into a single model that can reflect multiple levels of biological data. This is especially relevant for researches in economically-driven or similar fields, such as bioethanol production and other industrial capacities, where the ability to select targets for bioengineering approaches that maximize the desired effect (e.g. improving ethanol production) while minimizing undesired side-effects (e.g. affecting unrelated pathways and/or other phenotypes) can be of paramount importance to gain a competitive edge. By using our network model as a frame of reference to develop strains that are more robust to the effects of inhibitory compounds, such as pCA, we hope to drive innovation towards a more robust yeast strain that is capable of improved efficiency under the strenuous conditions imposed by industrial fermentation vats.

## Methods

### Yeast strain and cultivation conditions

The strain investigated in this study, *S. cerevisiae* SA-1, is an industrial strain obtained from Fermentec (Piracicaba, Brazil). Inoculum cultures were prepared from glycerol stocks stored at − 80 °C on a defined medium^129,130^, whose composition (in g L^−1^) is described as follows: (NH_4_)_2_SO_4_, 5.0; KH_2_PO_4_, 3.0; MgSO_4_.7H_2_O, 0.5; 1 mL L^−1^ trace element solution, 1 mL L^−1^ vitamin solution, and 25 g L^−1^ glucose. Cultures were grown overnight at 30 °C in a rotary shaker at 200 rpm. Chemostat cultivation with pCA was performed in a 2.0 L water jacket model Labfors 5 (Infors AG, Switzerland) with 1.0 L working volume, which was kept constant by a mechanical drain and a peristaltic pump. Throughout cultivation, both the culture vessel (0.5 L min^−1^) and the medium vessel (flow rate not measured) were purged with nitrogen gas to maintain anaerobic conditions. The agitation frequency was set at 800 rpm, the temperature was controlled at 30 °C, and the pH was adjusted to 5.0 using a controlled 2 M KOH solution. Pre-cultures for batch bioreactor cultivations were grown overnight in an orbital shaker at 30 °C and 200 rpm in 500 mL shake flasks containing 100 mL of the defined medium with 20 g L^−1^ starting glucose. The medium had the same composition as the preculture, except that Tween 80 and ergosterol were added at a final concentration of 0.01 g L^−1^ and 0.42 g L^−1^, respectively, to allow anaerobic growth. The batch phase was terminated after glucose depletion (monitored by a sharp drop in CO_2_ concentration in the exhaust gas), whereupon cultivation switched to continuous mode with the addition of fresh medium supplemented or not supplemented with 7 mM pCA. The dilution rate was set at 0.1 h^−1^ and the cultivation was assumed to be in a steady state when the dry weight of the culture and the specific carbon dioxide production rate varied by less than 2% for two volume changes during at least five residence times^131^.

The chemostat system was chosen due to its characteristics of maintaining physiological conditions in constant values among experiments, which is important when trying to isolate transcriptomic alterations that arise in response to a singular input (in our case, the presence of pCA), eliminating the effects of growth rates and other stochastic perturbations which may arise due to environmental conditions^132^.

### Analysis of extracellular metabolites

Cell dry mass concentration was determined by the gravimetric method^133^. Extracellular metabolite samples from the chemostat cultures were filtered through 0.2 µm syringe filters. Concentrations of residual carbon, ethanol, glycerol, and organic acids were quantified by high-performance liquid chromatography (HPLC)^134^, using a Prominence HPLC model (Shimadzu Corporation, Japan) and an HPX-87H analytical column (Bio-Rad Laboratories, USA) at 60 °C with 5 mM H_2_SO_4_ as mobile phase at 0.6 mL min^−1^. Ethanol concentrations were corrected for evaporation^135^ and pCA was quantified^136^ by using an HPLC and a C18 analytical column (Supelco Inc. model Waters Spherisorb ODS—25 µm, 250 mm × 4.6 mm) at 30 °C with 2% (v/v) acetic acid in ionized water (eluent A) and acetic acid 0.5% in ionized water and acetonitrile (50:50, v/v; eluent B) as mobile phase at 1.0 mL min^−1^ using a gradient program: from 10 to 15% B (10 min), 15% B isocratic (3 min), 15 to 25% B (7 min), 25 to 55% B (30 min), 55 to 100% B (1 min), 100% B isocratic (5 min), from 100 to 10% B (0.1 min). The total run time was 60 min, with a flow rate of 1.0 mL min^−1^ and an oven temperature of 30 °C. The injection volume for all samples was 10 μL. Monitoring was performed using a Shimadzu UV detector at wavelengths of 280 nm and 320 nm. Concentrations of compounds were calculated from calibration curves obtained from standard solutions^131^.

### RNA extraction and sequencing

RNA extraction was performed using the Direct-zol™ RNA MiniPrep kit (Zymo Research catalog no. R2051) following the manufacturer’s instructions. RNA samples were sequenced using BGISEQ-500, with each library generating approximately 24 M paired-end reads of 100 bp. Raw RNA-seq reads were filtered to remove adapter contamination and low-quality reads, with Trimmomatic^137^. Each sample was aligned against the R64-1-1 version of the *S. cerevisiae* reference genome, which is based on the S288C strain, with STAR v2.7.0^138^, using “—sjdbGTFfile,” “–quantMode GeneCounts”, “–twopassMode Basic” and the ENCODE guidelines for best practices of eukaryotic RNASeq^139^ as additional parameters. The corresponding gene annotation files and variant call files were also obtained for the same assembly. All genome data was obtained from Ensembl Fungi release 48^140^.

### Gene expression analysis and functional characterization

Differential gene expression was assessed by edgeR v.3.3^141^, using FDR ≤ 0.01 and |log2(FoldChange)| ≥ 0.5 as cutoffs for statistical significance. Gene expression in log2(CPM) scale was used to perform principal component analysis (PCA) and replicate similarity assessment to check the significance of biological duplicates. All downstream functional enrichment analyses were done using STRINGdb v.11^142^, and pathway perturbation analysis with Pathview API and GAGE v.2.38^143,144^. In both cases, an FDR cutoff of 0.01 was applied using the coding genome as background, and the Fold enrichment value was calculated based on the number of observed genes in comparison to the number of expected hits.

### Co-expressed gene cluster detection and hub gene identification

Co-expressed gene clusters were identified using an adaptation of the kNN-enhance method, which intensifies an existing network with node attributes^145^. The total protein–protein interaction (ppi) network from STRINGdb (v11) was converted into an undirected graph, where each node is a protein and the edges represent known interactions between them—only interactions with a total ppi_score ≥ 0.7 (high confidence) were considered for downstream analysis. Each node metadata information was enhanced with an extra attribute corresponding to the log2(foldChange) value of that protein, and for each pair of vertices connected by an edge, a second score was used (called foldChange_score). This metric was calculated by $1-norm\left({\left({X}_{i}-{X}_{j}\right)}_{2}\right)$ where ${X}_{i}$ is the foldChange for vertex 1, and ${X}_{j}$ is the foldChange for vertex 2, and $norm\left({\left({X}_{i}-{X}_{j}\right)}_{2}\right)$ is the normalized Euclidean distance between ${X}_{i}$ and ${X}_{j}$. Thus, values for foldChange_score varied from 1 (identical foldChange scores) to 0 (the largest foldChange difference between two nodes in the network).

Final edge weight scores were calculated by combining foldChange_score and ppi_score in a new “enhanced_score.” Co-expressed gene clusters were identified using MCL clustering^146,147^, applied to the attribute-enhanced network, with inflation hyper-parameter tuned to maximize modularity score (Q). For each of the identified clusters, it was also extracted genes that could serve as “local hubs” based on four different metrics: degree, betweenness, eigenvector and closeness.

BNFinder^148^ was used For the association between genes and phenotypical data, combining per-sample normalized gene expression (from RNASeq) with physiological data (derived from HPLC), and converted the observations into classes with the Sturges’ rule^149^.

A two-fold strategy was applied to generate cluster functional labels: the first was based on gene ontology enrichment classes, with the most significant enriched class (lowest FDR) that represented at least 50% of genes within the cluster; the second strategy was based on Bayesian inference of association with physiological data, with genes being able to be associated either positively or negatively with the changes in each measured metabolite.

### Short variant discovery

Short variants (SNPs and Indels) were identified using the GATK4 pipeline, in accordance with the best practices for RNASeq short variant discovery^74,75^. Aligned RNA/seq reads in BAM format were used as input, as well as GFF and VCF files for R64-1-1 annotation obtained from Ensembl. Short variant impact was estimated using Ensembl Variant Effect Predictor^150^⁠.

### Multi-omics network assembly

A graph-based approach was used to integrate all data layers (gene expression, co-expressed cluster hubs, pathway impact and nucleotide variants) into a unified network model using NetworkX^151^. The information for each layer was re-structured and merged with the others in a way that for every pair of vertices υ and ν, the first vertex (υ) represents a gene and the second vertex (ν) represents the characteristic associated to that gene (a pathway, phenotype or mutation). The weight of the edge defined by (υ,ν) was assigned according to the relationship between the expression change of the gene and alteration on the pathway/phenotype: + 1 for direct relationships (the direction of the gene fold change is in the same direction of the altered pathway/phenotype, i.e. both upregulated), − 1 for inverse relationships (the direction of the gene fold change is in the opposed direction of the altered pathway/phenotype, i.e. one upregulated while the other is downregulated) or 0 for neutral relationships (in the case of nucleotide variants). Directionality of the network was established in accordance with the following structure: variant → gene → pathway/phenotype, to reflect the idea that: “a nucleotide variant may affect the gene function, leading downstream alterations”.

### Ethical approval

This article does not contain any studies with human participants or animals performed by any of the authors.

## Supplementary Information

Supplementary Legends.

Supplementary Information 2.

## Data Availability

Raw sequencing files, scripts and all supplementary data are available at https://labis.cbmeg.unicamp.br/labis/publicacoes/71-sa1-pcoumaric, fastq reads were deposited on Sequence Read Archive with the following accession codes: SRR15944188 (DP06); SRR15944187 (DP07); SRR15944185 (DP09); SRR15944186 (DP19). Additionally, all files can also be accessed via Unicamp’s Research Data Repository via DOI code 10.25824/redu.
