# Supplementary material for: Multi-omics network model reveals key genes associated with p-coumaric acid stress response in an industrial yeast strain
Source: Sci Rep. 2022 Dec 28;12:22466. doi: 10.1038/s41598-022-26843-2 (PMC9797568; doi:10.1038/s41598-022-26843-2)
Supplement: Supplementary file 1 — Supplementary Legends. [file 41598_2022_26843_MOESM1_ESM.pdf]

Table S1 - Phenotypical data

Phenotypical data measured during fermentation processes of SA1 yeasts under control and p-Counaric acid stress conditions. Specific rates (q) are given in mmol g-1 h-1, μ in h-1, yeast biomass (X) in g DW L-1, conversion factor of substrate into biomass (YX/S) in g DW g glucose-1, ethanol yield (YEth/S) in g ethanol-1 g glucose-1, and C recovery in (%). The μ value represents the growth rate measured during the batch phase before the steady-state is achieved. Data is the average values of duplicate experiments ± deviation of the mean.

Table S2 - Differential expression

Differential gene expression measured between p-Coumarid acid stress vs. control conditions for SA1 yeasts. The expression values, both for average expression and per-sample expression, are represented in log2(CounterPerMillion) scale and fold-change scores are in log2(FoldChange) scale.

Table S3 - Pathway impact

Predicted pathway alterations based on gene-expression changes for SA1 yeasts under p-Coumaric acid stress. Each line represents one KEGG pathway with predicted perturbation in the dataset. For each entry, there is an associated p-value for upregulation (p.up) and one for downregulation (p.dn), an overall perturbation p-value (p-val) and adjusted p-value by benjamini-hochberg false discovery rate procedure (FDR or q-val). Enrichment rations were calculated based on the number of observed genes in the pathway (set.size) vs. the number of expected genes based on the coding genome (expected hits).

Table S4 - Cluster membership

Cluster membership for each differentially expressed gene identified based on node-attributed-enhanced MCL. The columns represent the gene name, the overall log2(FoldChange) observed for that gene, the cluster associated with that gene and the total number of genes in the cluster.

Table S5 - Clusters expression

Overall distribution of gene expression changes (in log2(FoldChange)) for each MCL-defined cluster. Boxplot statistics for log2(FoldChange) values observed for genes in each cluster.

Table S6 - Clusters enrichment

Expanded list of functional enrichment classes calculated for each MCL-defined cluster. Per cluster enrichment analysis of terms, including the ratio of genes in enriched classes in comparison to number of genes in each cluster.

Table S7 - Clusters phenotypes

Gene-Phenotype association table generated by Bayesian inference based on changes observed in gene expression and phenotypical data. The first columns shows the gene in the cluster, the second column the direction of interaction (“+” is direct; “-” is inverse) and the most-likely associated phenotype.

Table S8 - Hub gene list
List of genes that were identified as hubs in the co-expressed clusters. Each line shows a specific gene, with their cluster membership and uniprot keyword annotation.

Table S9 - Short-variant data
Short-variant, SNPs and INDELs, data (in comparison to S288C lab-strain) obtained through RNA-seq data. This table shows the Ensembl's variant-effect predictor output for each SNP identified in SA-1 transcriptome in comparison with the S288C reference strain (R64-1-1).

Table S10 - Multi-omics network
Network model for the multi-omics integrated graph containing pathway impact, phenotype association and structural variants. Table in edge list format with the nodes and their associations found in our multi-omics network. The first node (V1) is always a gene and the second node (V2) is a feature associated with that gene. Edge weights represent different types of interactions, which can be either direct (+), inverse (-) or undetermined (0).
